# Traumatic Brain Injury-Induced White Matter Disruption and Its Impact on Information Processing Speed—Theoretical and Clinical Implications: A Selective Review

**DOI:** 10.3390/jcm15114020

**Published:** 2026-05-22

**Authors:** Bar Lambez, Eli Vakil

**Affiliations:** 1 Department of Psychology, Loewenstein Rehabilitation Center, Ra’anana 43100, Israel; 2 Department of Behavioral Sciences, Ruppin Academic Center, Emeq Hefer 40250, Israel; 3 Department of Psychology and Gonda Multidisciplinary Brain Research Center, Bar-Ilan University, Ramat-Gan 52900, Israel

**Keywords:** TBI, processing speed, white matter, cognitive assessment

## Abstract

Recent paradigms in traumatic brain injury have transitioned from focal-lesion models to an emphasis on diffuse axonal injury and white matter disruption as the primary drivers of cognitive morbidity. This selective review frames information processing speed as the functional signature of this connectivity loss. While processing speed is often theorized as a “cognitive bottleneck” that constrains higher-order functions, we identify critical methodological and conceptual pitfalls in the existing literature. Specifically, we argue that current research is frequently confounded by: (1) measurement impurity, where tasks like the SDMT and TMT-B recruit executive and mnemonic variance; (2) circularity, where speed measures are used to predict time-dependent outcomes; and (3) the neglect of speed–accuracy trade-offs, which may mask volitional cautiousness as neurobiological incapacity. To resolve these challenges, we offer evidence-based recommendations for the clinical setting, including the integration of construct-pure chronometric measures and dual-scoring protocols. We conclude that because white matter integrity functions as a rate-limiting substrate, processing speed must be prioritized as a primary target in early neurorehabilitation. By isolating processing speed from focal-driven deficits, clinicians can more accurately map the path from microstructural disruption to functional recovery. Recognizing this infrastructure is essential to understanding the full scope of cognitive consequences.

## 1. Introduction

Processing speed deficits are among the most common, persistent, and functionally consequential cognitive sequelae of traumatic brain injury (TBI). Meta-analytic evidence documents impairments across the full severity spectrum and through all recovery phases, with prevalence reaching approximately 57% in chronic moderate-to-severe cases [1] and large effect sizes persisting well into the chronic period after severe injury [2]. These deficits have been recognized since the earliest systematic neuropsychological investigations of head injury: Gronwall [3] demonstrated reduced information processing capacity after concussion using the Paced Auditory Serial Addition Test (PASAT), and subsequent work confirmed that slowed processing is among the most sensitive neuropsychological markers of TBI across the severity spectrum [4,5,6]. Because processing speed constrains everyday activities and vocational outcomes, its assessment is a clinical priority that figures centrally in consensus rehabilitation guidelines [7].

The neuroanatomical basis for this slowing is diffuse axonal injury (DAI), the widespread microscopic shearing and stretching of axons caused by rotational acceleration–deceleration forces that characterize TBI as a neuropathological entity [8,9,10]. Unlike focal cortical contusions, which damage circumscribed regions and produce domain-specific deficits, DAI targets the white matter infrastructure that supports rapid communication between brain regions, making TBI fundamentally a disorder of connectivity [11]. Diffusion tensor imaging (DTI) studies have confirmed that fractional anisotropy (FA) reductions across white matter tracts correlate with processing speed deficits across the TBI severity spectrum [12,13], and more recent biophysically informed diffusion models such as Neurite Orientation Dispersion and Density Imaging (NODDI) reveal neurite density and orientation dispersion changes that relate to processing speed in ways that conventional DTI metrics miss [14,15]. The white matter–processing speed relationship is not unique to TBI, as analogous associations appear in normal aging [16,17,18,19,20] and multiple sclerosis [21,22]. However, in TBI there is particular importance in understanding the contribution of processing speed impairments, to better capture the consequences of damage that extend beyond the focal-lesion site, which is where most work has traditionally focused. Clarifying this relationship in TBI can therefore sharpen our understanding of how diffuse white matter disruption shapes cognitive and functional outcomes.

The theoretical position advanced in this review is that processing speed functions not merely as one impaired cognitive domain among many after TBI but as a proximal constraint on higher-order cognition: compromised white matter reduces the rate of neural information transfer, limiting the throughput available for working memory, executive control, and episodic encoding. Salthouse [23,24] formalized this bottleneck concept in the aging literature, and TBI research provides strong converging support. Structural Equation Modeling and covariance designs in TBI cohorts have demonstrated that processing speed mediates the relationship between injury severity and adaptive (social and occupational) functioning [25] and that controlling for elementary speed (e.g., simple or choice reaction time) attenuates or eliminates group differences on more demanding timed tasks [26]. While such findings are somewhat trivial when restricted to explicitly time-limited tasks, they nevertheless support the broader bottleneck account developed by Salthouse [23,24], in which reduced processing rate constrains higher-order cognition even when performance is not overtly speeded. These patterns are consistent with a model in which diffuse white matter pathology creates a fundamental processing-rate limitation that cascades into diverse cognitive deficits.

A critical but underappreciated complication is that the tests or tasks used to measure processing speed after TBI and other disorders do not all assess the same construct. More specifically, the various tasks used in the literature to measure processing speed do indeed all contain a component that is sensitive to processing speed. The problem is that most of these tasks are not pure, in the sense that processing speed is assessed on the basis of a cognitive task—such as visual scanning (e.g., WAIS-IV Processing Speed Index—PSI) or executive functions (e.g., Trail Making Tests—TMTs) [27] (see Table 1). Therefore, when a deficit is found in performance on such a task, it does indeed reflect a deficit in processing speed but most likely also a deficit in the additional cognitive process required to perform the task.

This measurement heterogeneity has received insufficient attention in previous studies, yet it has implications for how both neuroimaging and behavioral findings should be interpreted.

This selective narrative review starts with Section 2 with an overview of the definition of processing speed and then a synthesis of three converging lines of evidence: neuroimaging evidence that TBI damages white matter (Section 3), behavioral and imaging evidence linking this damage to processing speed deficits, and evidence that processing speed constrains higher-order cognitive and functional outcomes. Section 4 provides a task analysis of commonly used processing speed neuropsychological tests, examining what each measures beyond speed per se—a necessary foundation for interpreting the mediation evidence that follows. The Discussion Section integrates these threads and offers assessment recommendations grounded in the reviewed evidence. The literature was identified through PubMed, PsycINFO, and Google Scholar from 1990 to February 2026. Search terms included combinations of traumatic brain injury, diffuse axonal injury, white matter, processing speed, cognitive measures, reaction time, Symbol Digit Modalities Test, Trail Making Test, and paced auditory serial addition test. We prioritized peer-reviewed empirical studies, meta-analyses, and theoretically important papers and excluded studies that did not bear directly on the white matter–processing speed relationship. Because this is a selective narrative review, the search was not exhaustive, and study selection was guided by conceptual relevance rather than formal inclusion criteria.

## 2. Processing Speed: Construct Definition and Measurement Overview

Processing speed refers to the efficiency with which basic cognitive operations are executed—perceiving stimuli, making decisions, and producing responses [28,29]. It is not reducible to simple reaction time or motor speed; rather, it indexes the rate at which coordinated neural operations translate stimulus input into behavioral output. Processing speed shows robust associations with general intellectual ability [30,31], and large-sample evidence indicates that age-related effects on general cognition are substantially transmitted through slowing [32], reinforcing the theoretical position outlined in the Introduction Section that speed functions as a foundational cognitive capacity.

Applying the processing speed framework to TBI requires two caveats that have been recognized since the earliest systematic reaction time studies of head injury [4,33]. First, whether TBI produces uniform global slowing or differential slowing that disproportionately affects specific processes remains debated: Bashore and Ridderinkhof [34] argued for differential slowing using reaction time and P300 latency data, whereas Felmingham et al. [26] found a pattern more consistent with general slowing. Second, observed slowness can reflect both reduced processing capacity and volitional cautiousness, whereby patients adopt conservative response strategies to preserve accuracy, that is, a speed–accuracy trade-off [35]. Elevated reaction times may therefore overestimate true neurobiological slowing, and careful task selection is essential.

The dependence of processing speed on white matter integrity is well established across populations, providing a neurobiological foundation for understanding why TBI, a condition that preferentially damages white matter, produces such pervasive speed deficits. Diffusion imaging studies in healthy aging consistently show that age-related white matter degradation predicts processing speed decline [16,17,18,19], and in multiple sclerosis, demyelination-driven white matter damage similarly predicts Symbol Digit Modalities Test (SDMT) deficits [21,22]. These cross-population findings establish a general principle: processing speed is constrained by white matter integrity regardless of the cause of damage. This principle provides the mechanistic rationale for the TBI-specific evidence reviewed in the following section, where DAI produces widespread white matter disruption and correspondingly pervasive processing speed deficits. These cross-population findings show that processing speed is sensitive to white matter integrity in several disorders, but TBI provides a distinct case because the primary injury is mechanical axonal disruption rather than gradual degeneration or inflammatory demyelination.

## 3. White Matter Integrity, Processing Speed, and Higher-Order Cognition in TBI

The link between white matter microstructure and processing speed is not unique to TBI, as shown in the last section. More broadly, the hypothesis that processing speed constrains higher-order cognition originated in the aging literature: Salthouse [23,24] proposed that slowing creates a bottleneck that cascades into working memory, executive, and episodic memory deficits. Cross-population work has supported this. Lee et al. [36] showed processing speed-mediated age-related episodic memory decline, DeLuca et al. [37] found speed deficits primary to working memory impairments in multiple sclerosis, and Killingsworth et al. [38] extended the mediating role to prospective memory in older adults. These converging findings establish a general principle: processing speed is constrained by white matter integrity regardless of the cause of damage, and speed in turn constrains downstream cognition. TBI, with its pervasive white matter disruption and frequent speed deficits, provides a critical test of whether this bottleneck mechanism operates in TBI. Although aging, multiple sclerosis, and TBI all show associations between white matter integrity and processing speed, the neuropathological substrates are not interchangeable. In aging, white matter decline is typically gradual and multifactorial; in multiple sclerosis, slowed processing is linked primarily to inflammatory demyelination and lesion dissemination; in TBI, the dominant mechanism is diffuse axonal injury caused by mechanical strain, often accompanied by focal lesions and evolving secondary degeneration. The bottleneck model therefore applies across conditions at the level of disrupted communication efficiency, but the biological route to that disruption differs substantially. The following sections examine the evidence.

### 3.1. Neuroimaging Evidence of White Matter Damage in TBI

Conventional DTI reveals the in vivo footprint of DAI: widespread reductions in FA and increases in mean diffusivity (MD) across major white matter tracts, reflecting disrupted axonal organization and coherence. More biologically specific diffusion models extend this picture. Longitudinal work using NODDI in the TRACK-TBI cohort demonstrated early elevations of free-water fraction consistent with vasogenic edema, followed by progressive reductions in neurite density index (NDI) concentrated in posterior periventricular tracts—a temporal sequence suggesting ongoing axonal degeneration beyond the acute injury phase [15]. Bourke et al. [14] reported spatially heterogeneous NDI reductions and altered orientation dispersion in chronic moderate-to-severe TBI, patterns not captured by FA alone. Grassi et al. [39] documented evolving DTI abnormalities in the corpus callosum and superior longitudinal fasciculi across 2, 6, and 12 months after moderate-to-severe TBI with DAI, confirming that white matter pathology is not static but continues to change well beyond the acute phase. These imaging studies establish that TBI produces measurable, widespread, and temporally evolving white matter disruption; the question of whether this structural damage maps specifically onto processing speed deficits is addressed next.

### 3.2. White Matter Damage and Processing Speed Deficits in TBI

The convergent literature links white matter microstructure to processing speed after TBI, and the evidence can be organized around three themes: the breadth of the association, its anatomical specificity, and its prognostic utility [13,40]. The earliest DTI studies established that the white matter–processing speed link in TBI is not confined to particular tracts but extends across the brain’s structural connectome. Kraus et al. [13] demonstrated that FA reductions across widespread tracts correlated with cognitive performance, including processing speed, across the severity spectrum. Kinnunen et al. [12], using tract-based spatial statistics, confirmed that diffuse FA reductions were associated with slower processing speed alongside memory and executive deficits, a pattern consistent with domain-general cognitive consequences of widespread axonal injury. Wallace et al. [40] meta-analytically aggregated DTI–cognition studies in adults with TBI and confirmed reliable, cross-study associations between white matter integrity and processing speed.

Subsequent work identified which specific tracts are the most critical. Kourtidou et al. [41] showed that FA in the corpus callosum and centrum semiovale related to SDMT and TMT-B performance, implicating interhemispheric and long association fibers in timed processing. Owens et al. [42] extended this to the superior longitudinal fasciculus and cingulum, and Hanks et al. [43] demonstrated that both global and regional FA predicted SDMT scores. Spitz et al. [44] reported that FA correlated with both injury severity and cognitive outcomes, reinforcing the dose–response relationship between structural damage and functional impairment. The consistent emergence of the corpus callosum across these studies is noteworthy; as the brain’s largest commissural tract, it supports interhemispheric communication that is critical to the rapid coordination of distributed processing networks, making it a structural bottleneck whose disruption would be expected to slow information transfer broadly.

At the mild end of the severity spectrum, microstructural measures retain prognostic value that conventional imaging lacks. Niogi et al. [45] showed that DTI-derived abnormalities correlated with reaction time in mild TBI, while standard lesion counts did not, demonstrating that microstructural disruption, not macroscopic damage, drives speed deficits. Bai et al. [46] introduced a lesion-load DTI approach showing that acute FA reductions in strategically located tracts predict which mild-TBI patients will develop persistent processing speed deficits at 6–12 months, an early structural signature of chronic slowing with clear clinical utility for identifying patients who need sustained follow-up. Advanced diffusion models further strengthen these associations: Palacios et al. and Bourke et al. [14,15] found that NODDI-derived neurite density indices relate more specifically to processing speed than FA alone and longitudinal NDI declines predict enduring cognitive slowing.

### 3.3. Processing Speed Deficits in TBI: Behavioral Evidence

Behavioral research has characterized processing speed deficits as a core cognitive consequence of TBI since the earliest neuropsychological investigations of head injury. Gronwall and Wrightson [47] used the PASAT to demonstrate that information processing capacity was reduced after concussion and that recovery of processing speed predicted return to work, establishing speed as both a sensitive and functionally relevant marker. Classic reaction time studies confirmed robust slowing across paradigms of increasing complexity: Shore [33] demonstrated disproportionate slowing on four-choice visual reaction time relative to simple reaction time, with choice RT better discriminating severity subgroups defined by coma duration; Stuss et al. [48] extended these findings using simple RT, choice RT, and a focused-attention paradigm requiring inhibition of redundant information, showing that both TBI patients and older adults were selectively impaired on the more demanding conditions. Ponsford and Kinsella [6], using a comprehensive battery that included simple and choice RT, the SDMT, Stroop color naming and word reading, and the PASAT, demonstrated that slowed information processing was the most consistent attentional deficit after severe TBI, more prominent than problems with selective, sustained, or supervisory attention. Felmingham et al. [26] provided evidence that the speed deficit is tied specifically to diffuse axonal pathology: in a TBI sample (enriched for DAI), patients were significantly slower than both mixed-injury and control groups on basic speed tasks, with deficits persisting regardless of injury severity, age, or time since injury. This study is particularly important because it used relatively pure speed measures, simple and choice reaction time, alongside more complex tests, enabling the demonstration that group differences on complex speed tasks were attenuated after controlling for elementary processing rate, thereby minimizing the interpretive confounds associated with complex clinical tests.

Fong et al. [49] administered six processing speed measures spanning a complexity continuum, simple reaction time, movement time, choice reaction time, the SDMT, TMT-A, and TMT-B, to participants with TBI and showed that the measures diverged substantially in sensitivity and cognitive profile, with simple reaction time proving the most sensitive discriminator; this confirms that measurement choice matters, a point elaborated in the task analysis of Section 4.

### 3.4. Processing Speed as a Proximal Constraint on Higher-Order Cognition

The imaging and behavioral evidence reviewed above establishes that TBI damages white matter and that this damage produces processing speed deficits. A further question is whether these speed deficits merely coexist with other cognitive impairments or actively constrain them, functioning as a bottleneck through which higher-order deficits are transmitted, as Salthouse [23,24] proposed in the aging literature.

A substantial body of TBI-specific research has been interpreted as supporting the bottleneck model, though the strength of this evidence depends critically on whether outcome tasks are themselves speed-dependent. Rassovsky et al. [25], using Structural Equation Modeling in 87 moderate-to-severe TBI patients, demonstrated that processing speed, indexed by WAIS-R Digit Symbol and Symbol Search (see Section 4 for construct-purity considerations), but not verbal memory, mediated the relationship between injury severity and adaptive everyday functioning 12 months post-injury. Because adaptive functioning is not a timed laboratory measure, this finding offers partial support for the bottleneck model; however, because Digit Symbol and Symbol Search are themselves multifactorial (see Section 4), the mediating role of “speed” may partly reflect shared non-speed variance between the mediator and injury-related cognitive impairment rather than a pure speed bottleneck. Felmingham et al. [26] showed that in patients with severe TBI classified as having predominantly diffuse injury on MRI, controlling for basic reaction time speed eliminated group differences as measured with SDMT and Digit Symbol. Because the covariate was a relatively pure RT measure, this pattern is consistent with a bottleneck account. However, because the outcome tasks are themselves time-pressured, the attenuation of group differences could reflect shared speed-dependent variance rather than evidence that elementary speed constrains non-speed cognitive processes. Demonstrating a genuine capacity bottleneck, as Salthouse [23,24] did in aging, would require showing that basic RT predicts performance on untimed complex tasks, evidence that the current TBI literature largely lacks. Willmott et al. [50], using the same research group’s paradigm, similarly found that slowed processing speed, indexed by simple RT measures, rather than working memory deficits, accounted for attentional impairments after TBI, though the same caveat applies if the attention measures used were themselves timed.

Dymowski et al. [51] extended these findings across multiple executive tasks using covariance designs, demonstrating that processing speed was associated with deficits in selective attention, response inhibition, and mental flexibility. Wilson et al. [52] found that processing speed produced the largest effect sizes among cognitive predictors of functional outcome in CENTER-TBI. The convergence across analytic approaches, Structural Equation Modeling [25], analysis of covariance [26,50,51], and large-scale cohort prediction [52], has been taken as evidence that the bottleneck is genuine. However, because most of these studies used speed-dependent outcome tasks, methodological convergence across analytic techniques does not resolve the interpretive ambiguity identified above: if both the mediator and the outcome are sensitive to slowness, finding that speed “accounts for” higher-order deficits may reflect shared measurement properties rather than a true cognitive bottleneck. This distinction is precisely what a critical review must flag, though the magnitude of the bottleneck effect depends on measurement choices, as discussed in Section 4. This bottleneck model has boundary conditions: processing speed does not fully account for all post-TBI cognitive deficits, particularly on untimed tasks and certain executive subprocesses; but these qualifications, along with implications for measurement purity, are addressed in the Discussion Section.

### 3.5. Recovery Trajectories

Processing speed recovery after TBI typically follows a nonlinear course rather than a simple monotonic return to baseline. Longitudinal analyses indicate an early phase of relatively rapid improvement followed by an inflection toward a prolonged plateau, a pattern broadly shared with other cognitive and motor functions, though processing speed may plateau earlier or at a lower level relative to premorbid functioning than domains such as verbal learning [53]. Rabinowitz et al. [54] modeled this trajectory and showed that processing speed gains are the largest in the first months after injury, after which improvement slows markedly. The shape and ceiling of this recovery appear constrained by the extent of underlying axonal disruption: greater DAI and persistent white matter microstructural abnormalities are associated not only with larger initial deficits but with slower, more limited recovery, thus suggesting that white matter integrity functions as a rate-limiting substrate for cognitive restitution.

The persistence of processing speed deficits is particularly evident following moderate-to-severe TBI. Long-term follow-up studies reveal that slowed processing speed can endure for years, in some cases a decade or more after injury. Draper and Ponsford [55] reported measurable processing speed impairment at ten years post-injury among survivors of moderate-to-severe TBI, and multisite cohort analyses such as the TBI Model Systems have documented enduring neuropsychological compromise at five years [56]. Spitz et al. [57] observed that processing speed remains impaired even after many other cognitive domains have stabilized, reinforcing the notion that processing speed is a uniquely persistent sequela of TBI. Carlozzi et al. [58] documented severity-graded processing speed impairments, with larger and longer-lasting deficits being observed at higher injury severities.

By contrast, outcomes after mild TBI generally follow a different time course. Meta-analytic and cohort studies detect small-to-moderate group effects for processing speed in the early post-injury period. For example, Frencham et al. [59] reported an effect size around *g* = 0.47, but these impairments are most often transient, resolving over days to weeks in most cases [60]. Nevertheless, a minority of individuals with mTBI show persistent post-concussive symptoms and measurable cognitive slowing beyond the expected recovery window; longitudinal work using more sensitive diffusion metrics suggests evolving microstructural changes in subgroups that map onto differential recovery trajectories, underscoring the need to stratify follow-up by both clinical severity and biomarker evidence of axonal injury.

## 4. Task Analysis of Processing Speed Tasks

As noted in the Introduction Section, tasks used to measure processing speed are not always pure measures of speed; they recruit additional processes beyond elementary information accrual. The following analysis details these non-speed demands for frequently used processing speed tests, examines motor measures and a work-simulation task (VCWS 7) that help separate peripheral motor speed from central cognitive speed, and reviews studies that administered multiple speed tests to the same TBI samples to determine whether these tests produce comparable results. Table 1 presents a selection of the most widely used processing speed tests, along with the additional cognitive processes that each test may engage beyond pure speed.

**Table 1:** Complexity of processing speed measures.

Test	Core Speed Demand	Additional Non-Speed Demands	Clinical Caution	Key Studies in This Review
Simple Reaction Time (SRT)	Elementary response latency	Minimal	Closest to pure speed	Bashore & Ridderinkhof [34]; Battistone et al. [35]; Felmingham et al. [26]; Fong et al. [49]; Mathias et al. [61]; Niogi et al. [45]; Stuss et al. [48]; Shore [33].
Choice Reaction Time (CRT)	Response latency with decision rule	Stimulus identification, response selection	Slightly more executive load	Bashore & Ridderinkhof [34]; Battistone et al. [35]; Stuss et al. [48]; Shore [33].
Movement Time (MT)	Motor execution	Motor output only	Helpful for separating motor from central slowing	Fong et al. [49]; Houlihan et al. [62]; Incoccia et al. [63].
Finger Tapping	Fine motor speed	Minimal	Best as a motor control measure	Fong et al. [49]; Christianson & Leathem, [64]; Horton [65]; Prigatano [66].
MMDT Placing [67]	Visual scanning, sequential tracking	Graphomotor, visuomotor coordination	Not a pure speed test	Fong et al. [49]; Desrosiers et al. [68].
TMT-A [27]	Set shifting plus speed	Executive control, working memory	Strongly multifactorial	Bai et al. [46]; Cullen et al. [69]; Sánchez-Cubillo et al. [70].
WAIS-IV Symbol Search [71]	Visual scanning	Perceptual decision making	Less contaminated than Coding	Donders & Strong [72]; Carlozzi et al. [58]; Rassovsky et al. [25]; WAIS-R version).
WAIS-IV Coding [71]	Rapid symbol–digit transcription	Graphomotor, associative learning	Strongly multifactorial	Kennedy et al. [73]; Carlozzi et al. [58]; Rassovsky et al. (WAIS-R Digit Symbol) [25].
WAIS-IV PSI Composite [71]	Composite speed index	Combines multiple demands	Interpret cautiously	Carlozzi et al. [58]; Donders & Strong [72]; Kennedy et al. [73]; Rassovsky et al. (WAIS-R PSI) [25].
SDMT [74]	Symbol–digit matching	Working memory, learning, scanning	Multifactorial despite frequent clinical use	Felmingham et al. [26]; Kourtidou et al. [41]; Owens et al. [42]; Hanks et al. [43]; Sandry et al. [75]; Patel et al. [76]; Berrigan et al. [77].
PASAT [3]	Rapid auditory processing	Working memory, arithmetic, anxiety	Highly multifactorial	Gronwall [3]; Gronwall & Wrightson [47]; Ponsford & Kinsella [6]; Sherman et al. [78]; Tombaugh [79]; Berrigan et al. [77]; Fisk & Archibald [80].
TMT-B [27]	Manual dexterity	Eye–hand coordination	Motor/perceptual, not pure speed	Kourtidou et al. [41]; Bai et al. [46]; Fong et al. [49]; Captain’s Log Trail Sequence B).
VCWS 7—Multilevel Sorting [81]	Work-simulation throughput	Motor, perceptual, executive demands	Functional, not a pure speed test	Fong et al. [49].
Composite/multitest batteries	Elementary response latency	Minimal	Closest to pure speed	Dymowski et al. [51]; Wilson et al. (CENTER-TBI) [52].

Note: PS = processing speed; SRT = simple reaction time; CRT = choice reaction time; MT = movement time; MMDT = Minnesota Manual Dexterity Test; TMT = Trail Making Test; PASAT = Paced Auditory Serial Addition Test; WAIS-IV PSI = Wechsler Adult Intelligence Scale–IV Processing Speed Index; SDMT = Symbol Digit Modalities Test; VCWS 7 = Valpar Component Work Sample 7.

### 4.1. Simple Reaction Time (SRT)

SRT paradigms provide the most construct-pure estimates of elementary processing speed, requiring only stimulus detection, a simple decision, and a prompt motor response. They minimize working memory, associative learning, and executive demands, making them the most direct probe of the information-accrual bottleneck [23,24]. Felmingham et al. [26], Mathias et al. [61], and Niogi et al. [45] showed that RT measures reveal deficits even when composite clinical indices are inconclusive. Despite their theoretical value, simple RT tasks remain uncommon in routine clinical assessment tools due to limited norms and perceived lack of ecological validity.

### 4.2. Choice Reaction Time

This task is based on the simple RT paradigm, but the required response is now contingent upon a specific condition, for example, responding only when a green stimulus precedes the target and withholding the response when a red stimulus appears. This task, therefore, is no longer solely dependent on response speed; it additionally requires a decision-making process regarding whether the condition for responding has been met which is, in essence, an executive process. This task has been found to be sensitive to brain injury [33,48]. Bashore and Ridderinkhof and Battistone et al. [34,35] used choice RT paradigms to separate capacity limits from strategic cautiousness (see Section 2).

### 4.3. Trail Making Test Parts A and B 

TMT-A [27] indexes psychomotor efficiency, visuospatial scanning, and speeded visuomotor coordination through sequential number connecting. TMT-B [27] adds alternation between digits and letters, substantially increasing demands for cognitive flexibility, set shifting, and executive control. Decomposition studies confirm that TMT-A loads mainly on visuo-perceptual abilities with modest working memory contributions, while TMT-B reflects primarily working memory and secondarily task switching—executive function [70,82].

Because TMT-B recruits executive processes (e.g., set shifting and cognitive flexibility) in addition to speed, a deficit on this task cannot be attributed exclusively to processing speed, consistent with the general caution that applies throughout this review regarding the interpretation of cognitively loaded speed measures. Derived scores can help: the B−A difference provides a relatively pure indicator of executive control [70], and the B/A ratio correlates specifically with set-switching cost [82]. Corrigan and Hinkeldey [83] found that the difference score correlated with intelligence and severity of impairment; Lezak [29] recommended the B/A ratio for isolating executive from speed components; Greenstein et al. [84] showed that the A–B relationship provides additional information about attentional structure in developmental norms; and Christidi et al. [85] demonstrated that derived scores minimize demographic confounds. However, Martin et al. [86] found that the B/A ratio lacked sensitivity to injury severity in TBI, suggesting that clinicians should not rely on it alone.

Both parts have been linked to white matter integrity in TBI [41,46], and TMT-A contributes to predicting functional outcomes such as return to driving [69]. Fong et al. [49] used a computerized variant of Part B (Captain’s Log “Trail Sequence B”) in which mouse-driven selection replaces graphomotor drawing, reducing the motor component while preserving executive demands.

### 4.4. Paced Auditory Serial Addition Test—PASAT 

The PASAT [3] presents externally paced single digits that must be continuously added to the immediately preceding digit. Although developed to measure processing speed after concussion, it is now recognized as multifactorial [79]. Performance depends on at least four non-speed components: (1) working memory updating and sustained attention, as examinees must maintain, compute, and respond before the next stimulus arrives [80]; (2) arithmetic skill, which accounts for substantial PASAT variance independently of attention [78]; (3) strategic chunking, whereby examinees skip items to manage load, masking true capacity [80]; and (4) practice effects and test-induced anxiety [79]. Berrigan et al. [77] confirmed a multifactorial interpretation of both the PASAT and SDMT, concluding that this “facilitates their usefulness as screening measures for cognitive decline but prevents them from identifying which specific cognitive functions are affected.” (p. 1).

### 4.5. WAIS-IV Processing Speed Index—PSI 

The PSI [71] combines two subtests with overlapping (i.e., speed) but distinct demands. Coding requires symbol–digit transcription under time pressure, recruiting graphomotor execution, incidental associative learning, visual scanning, and sustained attention. The graphomotor component is substantial: examinees with motor impairment may score poorly for reasons unrelated to central speed. Symbol Search requires scanning for a target symbol and marking present/absent, with simpler motor demands, minimal working memory, and no associative learning, making it a somewhat purer measure of visual scanning speed.

Kennedy et al. [73] decomposed WAIS-III PSI composite variance in a TBI sample: working memory explained 10%, motor speed (finger tapping) 3%, and TMT-B 26%, totaling 56%. Because Coding dominates the PSI, these results imply that perceptual–cognitive rather than motor demands drive Coding performance, though the decomposition applies to the composite, not Coding in isolation. Carlozzi et al. [58] and Donders and Strong [72] report strong PSI discriminant validity for moderate-to-severe TBI. Comparing Coding and Symbol Search subtest scores can help clinicians determine whether graphomotor impairment or perceptual scanning difficulty is the primary contributor to a low PSI. Once again, as can be seen, despite being defined as measures of processing speed, these tasks are in practice “contaminated” by additional cognitive processes.

### 4.6. Symbol Digit Modalities Test—SDMT 

The SDMT [74] requires rapid symbol–digit pairing using a reference key, in oral or written format. In addition to processing speed, it recruits sustained visual scanning, short-term memory, and either graphomotor execution (written) or verbal production (oral). The SDMT is the clinical test most frequently linked to white matter microstructure in this review: associations with corpus callosum and centrum semiovale FA [26,41,42,43]. Berrigan et al. [77] confirmed that the SDMT supports a multifactorial interpretation to which processing speed, working memory, and learning all contribute.

### 4.7. Motor and Vocational Speed Measures

Distinguishing central cognitive slowing from peripheral motor impairment is a basic step in interpreting slowed performance after TBI. Lezak [29] treats motor speed and dexterity measures as motor functions, rather than attention/processing speed tests, because they index output constraints that can contaminate timed cognitive scores. In a typical TBI battery, these measures therefore serve as controls: intact motor speed with impaired cognitively loaded speed tasks supports a central processing interpretation, whereas impairment on both suggests that motor slowing may contribute to the apparent processing speed deficit.

### 4.8. Finger Tapping, Halstead–Reitan 

This task measures motor speed and motor control [64]. Horton [65] reported that higher intelligence predicted better performance on most Halstead–Reitan measures [27] except dominant-hand finger tapping, consistent with a primarily motor interpretation. In TBI, Prigatano et al. [66] observed patients with severe TBI with normal or near-normal tapping speeds years post-injury despite altered fMRI activation patterns, suggesting behavioral preservation with neural compensation.

Movement time (MT) separates motor execution from stimulus evaluation/decision components of response latency. Houlihan et al. [62] showed that reaction time tracked stimulus evaluation demands, whereas MT tracked motor execution difficulty, supporting the clinical value of RT/MT decomposition when interpreting slowness. Dexterity tests such as the Minnesota Manual Dexterity Test (MMDT) index visuomotor coordination; Desrosiers et al. [68] reported acceptable-to-high test–retest reliability and validity via correlations with Box and Block and Purdue Pegboard measures. In TBI, these measures help determine whether poor performance on graphomotor-heavy timed tasks (e.g., Coding) reflects central slowing or dexterity limitations.

Within TBI samples, Fong et al. [49] found no group differences on finger tapping, MT, or MMDT despite clear deficits on simple RT and more complex speed tasks, supporting a central (not motor) source of slowing in their sample. Stuss et al. [48] similarly found greater impairment on choice than simple RT. The dissociation is not universal: Incoccia et al. [63] reported slower MT even in patients following severe TBI with good clinical motor recovery. Puopolo et al. [87] estimated substantial sensory–motor slowing in severe TBI, cautioning against assuming that motor speed is always preserved. Lastly, vocational work-simulation tasks (e.g., VCWS 7) can be useful for ecological questions about work capacity, but they are too multifactorial to isolate processing speed and are best treated as functional measures rather than speed indices.

### 4.9. Multitest Comparisons

Studies administering multiple speed measures within the same sample with individuals following TBI directly test whether these tasks are interchangeable. Fong et al. [49] compared 20 outpatients with moderate-to-severe TBI with 20 matched controls on SRT, movement time, choice reaction time, SDMT, TMT-A, and VCWS 7. Sensitivity varied sharply: significant group differences emerged on SRT, trail speed, and VCWS 7 but not on MT or MMDT. In logistic regression, SRT alone achieved 75% sensitivity in classifying TBI, rising to 90% when combined with MMDT, demonstrating that pairing a central-speed metric with a motor measure improves discrimination.

Kennedy et al. [73] decomposed the sources of variance in the WAIS-III PSI in a TBI sample and found that perceptual and executive processing (26% of PSI variance from TMT-B) dominated over motor speed (3% from finger tapping), indicating that the PSI reflects cognitive complexity far more than peripheral motor efficiency. Mathias et al. [61] complemented this study by showing that simple chronometric measures (SRT) relate more directly to corpus callosum white matter integrity than complex clinical composites do—consistently with the view that purer speed measures are more sensitive to the underlying structural pathology. Some studies bypass individual tests altogether.

Taken together, these comparisons reinforce a point made throughout this review: processing speed is a well-defined construct, but the tests commonly used to measure it are contaminated by additional cognitive and motor processes. This contamination, not ambiguity in the construct itself, explains why different “speed” measures yield discrepant results and why the choice of measure shapes both the magnitude of the observed TBI effect and the interpretation of imaging–behavior correlations and mediation models.

## 5. Discussion

The clinical and scientific understanding of TBI has historically focused on the implications of focal lesions, primarily lesions of the frontal lobes. This approach, rooted in classical neuropsychology, proved powerful for characterizing the consequences of penetrating injuries and cortical contusions but was poorly equipped to capture the effects of DAI, the widespread microscopic shearing of white matter that is now recognized as one of the defining neuropathological features of TBI [8,9,10]. Only in the last few decades has white matter damage been recognized as a significant driver of cognitive impairment in TBI. This shift was enabled by diffusion imaging, which allowed for in vivo visualization of microstructural disruption [12,13,15]. This has created a need to clarify the cognitive consequences of disrupted connectivity, which are not well explained by focal-lesion models. Damage to the corpus callosum, for instance, does not produce a classical cortical syndrome but rather disrupts interhemispheric communication, a type of connectivity loss that can slow the coordination of distributed networks without any single cortical region being destroyed. The purpose of this review was to highlight processing speed as a key cognitive consequence of connectivity damage. As a selective review, this manuscript emphasizes conceptual synthesis rather than exhaustive coverage, so relevant studies may not have been captured. The dimension of impairment is often overlooked in diagnostic and rehabilitation practice, where the focus is usually on domain-specific deficits tied to identifiable lesions. In standard neuropsychological assessment, processing speed is usually measured indirectly. Composite scores such as the SDMT or WAIS-IV PSI are treated as “speed” measures, but as reviewed in Section 4, they also reflect other processes. Processing speed is rarely assessed with more construct-pure tasks, and even when deficits are identified, they are not often treated as a primary target. The result is a clinical practice that systematically underestimates the contribution of connectivity-mediated slowing to the overall cognitive profile after TBI.

The evidence reviewed in Section 3 established that white matter damage in TBI is consistently associated with processing speed deficits. DTI studies demonstrate widespread FA reductions that correlate with speed across the severity spectrum [12,13,40], with particular anatomical convergence on the corpus callosum and long association tracts [41,42,43]. Advanced diffusion models such as NODDI reveal that neurite density indices relate to processing speed more specifically than conventional FA [14,15], and longitudinal data confirm that white matter pathology continues to evolve beyond the acute phase, with progressive NDI declines predicting enduring cognitive slowing.

Several groups have conceptualized processing speed as a bottleneck for higher-order cognitive processes. Slower processing is thought to contribute to difficulties in working memory, executive control, and everyday functioning. This bottleneck framework, first developed in the aging literature by Salthouse [23,24], has received convergent support in TBI through Structural Equation Modeling [25], covariance designs with pure speed measures [26,50,51], and large-scale cohort prediction [52].

As indicated by the review above, information processing speed has indeed been studied in populations of individuals following TBI. Nevertheless, our review identified several methodological concerns with studies in which researchers attempted to argue that the basis for some of the characteristic deficits associated with TBI lies in impaired information processing speed. The following are the difficulties identified across various articles that examined the effects of TBI on information processing speed and, on that basis, claimed that such impairment underlies other cognitive deficits.

First, tasks designed to assess information processing speed also engage additional cognitive processes. It is therefore unsurprising that these tasks predicted deficits in other cognitive domains. Most clinical speed tasks are inherently multifactorial, recruiting executive functions, working memory, and visuomotor coordination. Consequently, it is unsurprising that these “contaminated” tasks predict deficits in other cognitive domains, as the predictor and the outcome often share executive variance. Crucially, this implies that the bottleneck model cannot be generalized without qualification, as some cognitive tasks are clearly independent of time. For untimed or power-based assessments, processing speed may be entirely irrelevant, and the bottleneck framework, which holds most clearly for throughput-limited, time-dependent outcomes, may not apply. In such domains, alternative explanations, such as focal pathology, compensatory strategies, or distinct executive subprocesses, should be considered instead.

Second, many of the studies that use processing speed as a predictor also rely on outcome measures that are themselves time-dependent. In that setting, mediation may partly reflect shared timing demands rather than a true capacity bottleneck, so the apparent explanatory role of processing speed should be interpreted cautiously. Third, even when speed is clearly involved, observed slowing may reflect a strategic speed–accuracy trade-off rather than a fixed neurobiological limit. Patients with TBI may deliberately respond more slowly to preserve accuracy, which can make reaction time look like primary processing impairment when it may instead reflect a conservative response style. Future studies should therefore report accuracy alongside reaction time, examine response distributions, and, whenever possible, separate elementary speed from strategic cautiousness.

Future studies can reduce circularity by pairing a relatively pure elementary speed measure, such as simple reaction time, with untimed complex task such as the Wisconsin Card Sorting Test, measuring executive functioning, and by separating processing rate from accuracy and strategy. Useful designs would include longitudinal cohorts, preregistered mediation models, latent-variable approaches that model speed and executive variance separately, and task batteries that include both speeded and untimed outcomes. Ideally, speed should be measured with minimal executive load, while higher-order cognition should be assessed with tasks that are not themselves timed. Additionally, the white matter–processing speed relationship is also likely moderated by individual differences. Age at injury, cognitive reserve, lesion burden, psychiatric comorbidity, and post-TBI depression may all influence recovery trajectories and apparent processing speed. In particular, depression can independently slow processing and complicate interpretation of cognitive slowing, so future work should model these variables explicitly rather than treating TBI as a uniform condition.

Drawing on the theoretical review presented above, we propose a set of recommendations for optimizing the use of information processing speed assessments, with clinical implications for both diagnosis and intervention. First, include in the assessment a pure speed measure. Neuropsychological batteries should include a measure of elementary processing speed, simple or choice reaction time, as pure as possible, and use it as a baseline against which performance on more complex timed tasks is compared. If a patient is slow on both SRT and the SDMT, this is consistent with a central-speed bottleneck affecting all timed performance; if SRT is intact but the SDMT is impaired, the deficit more likely reflects the additional processes the SDMT recruits (e.g., associative learning, visual scanning, and graphomotor execution) rather than elementary processing rate. This approach targets the cognitive consequences of disrupted neural connectivity rather than the effects of focal cortical damage alone and aligns with evidence that RT measures detect impairment even when standard clinical composites appear normal [26,45].

Second, pair matched tasks of differing complexity. An effective diagnostic strategy is to select task pairs in which one indexes processing speed with minimal additional demands and the other is structurally identical but adds a specific higher-order component. The comparison between the two reveals whether the deficit extends beyond speed. The classic example is TMT-A versus TMT-B: TMT-A primarily requires visuomotor scanning speed, while TMT-B adds executive control and set-shifting demands [70,82]. If TMT-A is within normal limits but TMT-B is impaired, one can attribute the deficit with greater confidence to executive dysfunction rather than baseline speed. Greenstein et al. [84] demonstrated that the relationship between TMT-A and TMT-B provides structural information about attention beyond what either part yields alone, and Lezak [29] recommended examining the relationship between the two parts as a standard interpretive practice.

Third, a particularly important methodological contribution to the assessment of processing speed was introduced by Kaplan and colleagues [88] through the WAIS-R as a Neuropsychological Instrument (WAIS-R NI). Kaplan [89] proposed that timed subtests be scored twice: once under standard time-limited conditions and once without time constraints. The discrepancy between these two scores allows the examiner to determine the extent to which performance decrements reflect slowed processing per se, as opposed to a genuine impairment in the cognitive domain being measured. This dual-scoring approach is particularly valuable following traumatic brain injury, where psychomotor slowing may otherwise artificially depress scores on timed tasks and obscure intact cognitive abilities—a distinction with direct implications for both diagnosis and rehabilitation planning.

Fourth, examine speed–accuracy trade-offs. Observed slowness in TBI may partly reflect volitional cautiousness, a conservative response strategy adopted to preserve accuracy, rather than a genuine reduction in processing capacity [35]. If accuracy is preserved or elevated alongside slow responding, clinicians should consider that the patient is trading speed for accuracy, and reaction time alone may overestimate the true neurobiological deficit in processing speed.

The theoretical implication is that processing speed should be treated as a foundational constraint on downstream cognition, but the clinical implication is more limited and should be stated more cautiously. Current evidence most strongly supports the early identification and monitoring of processing speed deficits, preferably with more construct-pure chronometric measures, because these results can help clinicians distinguish central slowing from the non-speed demands embedded in common tests [57]. The evidence base for specific speed-targeted rehabilitation is less uniform than the assessment literature, so recommendations should be tied to guideline-level support rather than presented as settled fact.

Clinically, this means that processing speed should inform rehabilitation planning, pacing, and compensatory strategy selection, especially early after injury when recovery is the most dynamic [53,54]. INCOG 2.0 [90] supports attention and information-processing speed rehabilitation as a clinical target, but the most defensible recommendation is to combine assessment, monitoring, and tailored cognitive rehabilitation rather than to claim that speed-focused intervention alone is sufficient. Persistent slowing after moderate-to-severe TBI [55] may justify continued follow-up, environmental simplification, and task pacing, while more intensive restorative intervention is best reserved for patients who show measurable speed-related impairment on relatively pure measures. Accurate quantification of processing speed deficits should directly inform vocational rehabilitation, steering patients toward occupational roles in which speed of execution is not a core demand. Equally, clinicians and employers should recognize that extended time allowances for task completion represent a meaningful and evidence-grounded accommodation for this population.

This position implicitly equates cognitive capacity with processing speed, a logic analogous to defining computational power solely in terms of processing rate. When TBI damages the white matter connections that support rapid neural communication, it does not merely impair one cognitive domain among many, it degrades the infrastructure on which all time-dependent cognition relies. Recognizing this in both assessment and rehabilitation is essential to understanding the full scope of cognitive consequences after traumatic brain injury. In this sense, processing speed serves as the cognitive signature of connectivity loss, a measurable bridge between neuroanatomy and function in TBI.

## Data Availability

No data are included in this review paper.
